# CMOS based capacitive sensor matrix for characterizing and tracking of biological cells

**DOI:** 10.1038/s41598-022-18005-1

**Published:** 2022-08-16

**Authors:** Reda Abdelbaset, Yehia El-Sehrawy, Omar E. Morsy, Yehya H. Ghallab, Yehea Ismail

**Affiliations:** 1 Biomedical Engineering Department, Faculty of Engineering, Helwan University, Cairo, Egypt; 2 Center of Nanoelectronics and Devices (CND), The American University in Cairo (AUC) and Zewail City of Science and Technology, Cairo, Egypt

**Keywords:** Biomedical engineering, Biosensors

## Abstract

The characterization and tracking of biological cells using biosensors are necessary for many scientific fields, specifically cell culture monitoring. Capacitive sensors offer a great solution due to their ability to extract many features such as the biological cells' position, shape, and capacitance. Through this study, a CMOS-based biochip that consists of a matrix of capacitive sensors (CSM), utilizing a ring oscillator-based pixel readout circuit (PRC), is designed and simulated to track and characterize a single biological cell based on its aforementioned different features. The proposed biochip is simulated to characterize a single Hepatocellular carcinoma cell (HCC) and a single normal liver cell (NLC). COMSOL Multiphysics was used to extract the capacitance values of the HCC and NLC and test the CSM's performance at different distances from the analyte. The PRC's ability to detect the extracted capacitance values of the HCC and NLC is evaluated using Virtuoso Analog Design Environment. A novel algorithm is developed to animate and predict the location and shape of the tested biological cell depending on CSM's capacitance readings simultaneously using MATLAB R2022a script. The results of both models, the measured capacitance from CSM and the correlated frequency from the readout circuit, show the biochip's ability to characterize and distinguish between HCC and NLC.

## Introduction

Monitoring and visualizing the cells' movement and growth under a physical actuator or cell culture conditions are critical applications for many biological scientists^1^. Biosensors are widely used because of their ability to provide continuous, real-time physiological information via dynamic, noninvasive measurements of the physical properties of biological cells in biofluids, such as sweat, tears, saliva, and interstitial fluid^2^. In addition, they offer high accuracy, speed, portability, low cost, and low power consumption^2^. This distinguishes integrated chips with regards to such applications from the traditional methods such as image processing of microscopic images, which are more complex, lack portability, and are higher in cost^3^.

Furthermore, these traditional techniques are time-consuming because they require several steps for sample preparation. They are sometimes toxic, making them unsuitable for continuous reading and needing a more extensive area^4^.

Implementing biosensors based on Complementary Metal Oxide Semiconductor (CMOS) technologies offer high throughput^1^. CMOS technology has many other advantages, such as being able to fit a large number of sensors with their associated electronic circuitry to create a single Laboratory on Chip (LOC), reducing the time consumed in biological analyses such as DNA analysis^5^, cancer detection^6,7^, continuous glucose monitoring^8^ and neurochemical detection^9^.

Not only do CMOS-based capacitive sensors feature compactness, but they also feature high sensitivity for many biological applications^4^. Capacitive sensors rely on detecting a dielectric change above or between the capacitive electrodes. A dielectric change occurs due to the ionic cloud in the cell membrane when the cell is introduced above capacitive sensors^10^. This change is tiny and therefore requires measurement using a susceptible capacitive readout circuit.

Previous work in the field of CMOS-based capacitive sensors has focused on analyte characterization or imaging. Senevirathna, Bathiya Prashan, et al. developed a CMOS-based capacitive array to quantify cell proliferation and adhesion^11,12^. Nabovati et al. designed a CMOS-based capacitive array to monitor continuous adherent cell growth^13^. Couniot et al. designed a CMOS based capacitive array to detect a single bacteria cell^14^. Zhang et al. designed an imaging-based capacitive sensor for defect detection^15^. Laborde et al. developed a high-density capacitive sensor array to image microparticles and living cells^16^.

The purpose of this research, however, is to perform both characterization and imaging of biological cells using a high density bipolar capacitive sensor array, a high-frequency ring oscillator-based readout circuit, and a novel script to predict the shape of the tested cell using cubic spline interpolation. As a proof of concept, a novel capacitive-based biochip is suggested to locate, characterize, and predict the shape of a biological cell; the work focuses on the distinction between a single Hepatocellular carcinoma cell (HCC) and a single normal liver cell (NLC). The proposed biochip consists of four major parts, a 10 × 10 matrix of capacitive sensors (CSM), a pixel readout circuit (PRC), a microfluidic chamber, and a personal computer. Unlike previous work, the design for the CSM features a top ground plate to ensure that there is no noise interfering with the reading. The CSM is simulated using the finite element method (FEM), specifically COMSOL Multiphysics 5.5. The PRC, designed and simulated on Virtuoso Analog Design Environment—Cadence, uses a high-frequency ring oscillator-based readout circuit with a digital frequency output that can be measured using a counter, eliminating the need for an analog to digital converter (ADC). Operating at a high frequency improves the sensor's sensitivity, as smaller capacitance changes result in more significant changes in the output frequency. Consequently, the circuit's complexity and power consumption are low, making it appealing for high-scale manufacturing purposes. The computer is used to run a novel algorithm that locates, characterizes, and predicts the shape of the biological cell based on the capacitance readings from the CSM using MATLAB R2022a.

## Methodology

**Figure 1:**
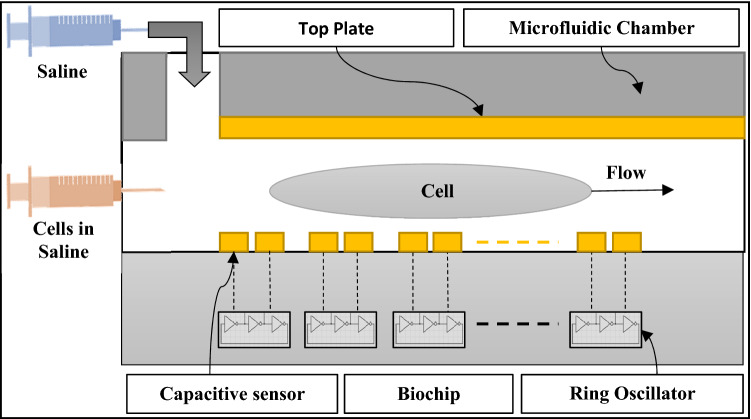
The block diagram of the proposed platform.

The objective of the proposed chip is imaging, locating, and characterizing the introduced biological cell based on its measured capacitance. As shown in Fig. 1, the proposed design consists of CSM, a microfluidic channel, PRC, and a personal computer. Each sensor acts as a terminal for generating an electric field and, simultaneously, as a sensor to sense the capacitance of the tested cell. The proposed microfluidic channel is intended to retain the top plate while also allowing injection and sample disposal. The functionality of the CSM, PRC and personal computer will be discussed in detail in the following sections.

### Capacitive sensors matrix (CSM)

The developed CSM consists of 10 × 10 sensors for sensing the capacitance of the biological cell within a medium. Each sensor consists of dual electrodes which act as a parallel plate capacitor with an intermediary dielectric layer in the form of the introduced cell. The capacitance reading for each sensor in the CSM is computed in order to monitor and identify the location of the introduced cell. These values are represented using the Maxwell capacitance matrix^17^. A Maxwell capacitance matrix provides a relation between voltages on a set of conductors to charges on the conductors^17^:

1 $$Q=C \cdot V$$

where Q is the charge, V is the potential difference relative to the ground, and C is the capacitance. COMSOL Multiphysics 5.5 is used to determine the most effective CSM configuration as well as to extract the capacitance values of saline, NLC, and HCC.

**Figure 2:**
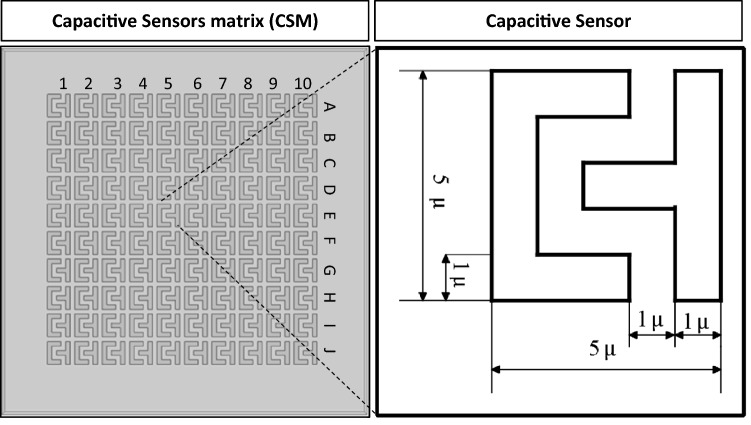
The geometry of the simulated CSM.

The CSM was simulated using the finite element method on COMSOL Multiphysics 5.5. The main parameters for the simulation are the proposed geometry configuration, material properties, and physics conditions. The geometry consists of CSM, a simulated cell, as shown in Fig. 2. Each sensor of the 10 × 10 CSM features dual bipolar electrodes with a width equal to 1 µm, a distance equal to 1 µm, and an area equal 5 × 5 µm^2^. However, this dimension is proposed due to TSMC 130 nm technology limitations at metal 7. In addition, we provide CSM without a passivation layer to improve sensitivity, as demonstrated by past laboratory experiments^18,19^. The proposed microchannel's height, width, and length are 40 µm, 80 µm, and 200 µm, respectively. A cylinder with a thickness of 10 μm and diameters of 20 μm and 50 μm is used to simulate the biological cells (NLC and HCC)^20–24^. As illustrated in Fig. 1, the proposed microfluidic channel has two inlets and one output. One inlet allows for the injection of biological cells suspended in a medium that has been evaluated (saline). Another inlet is intended to drive the injected cells to be as close to the CSM in order to increase the sensitivity of the proposed platform. The relative permittivity of the medium (saline) is 78.69, while the relative permittivity of the biological particles (NLC and HCC) is 53 and 58.5, respectively. The permittivity, on the other hand, is necessary to model the Maxwell capacitance matrix.

Any object with dielectric contrast in the neighborhood of the sensor will change the capacitive coupling between the electrodes. In order to determine the sensor activation configuration that maximizes the sensed capacitance, two configurations are proposed: with/without the ground top plate.

**Figure 3:**
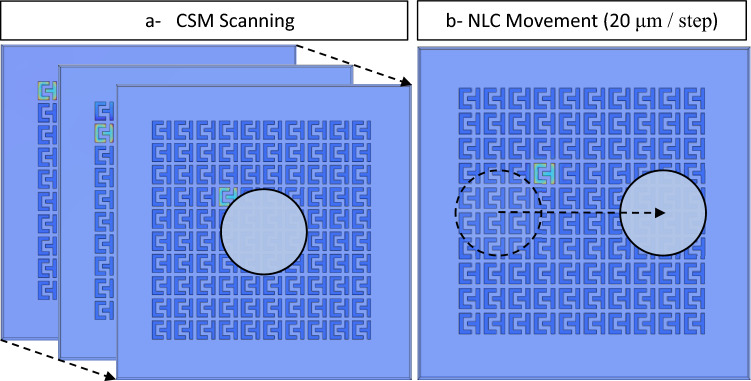
Simulation setup: (**a**) CSM scanning, (**b**) cell manipulation.

The simulation setup is portrayed in Fig. 3, which includes two phases: CSM scanning and cell manipulation. As shown in Fig. 3a, all sensors are activated sequentially in order to generate one frame at each step of cell movement. This is achieved by applying the Stationary Source Sweep study. The Stationary Source Sweep study generates default output nodes for three variants of the capacitance matrix resulting in the inverse Maxwell capacitance matrix. The electrostatic module is chosen to solve the capacitance matrix equations. The manipulation of the tested biological cells is simulated using deformed geometry and the parametric sweep using a movement rate equal to 20 μm/step, as shown in Fig. 3b.

### Pixel readout circuit (PRC)

**Figure 4:**
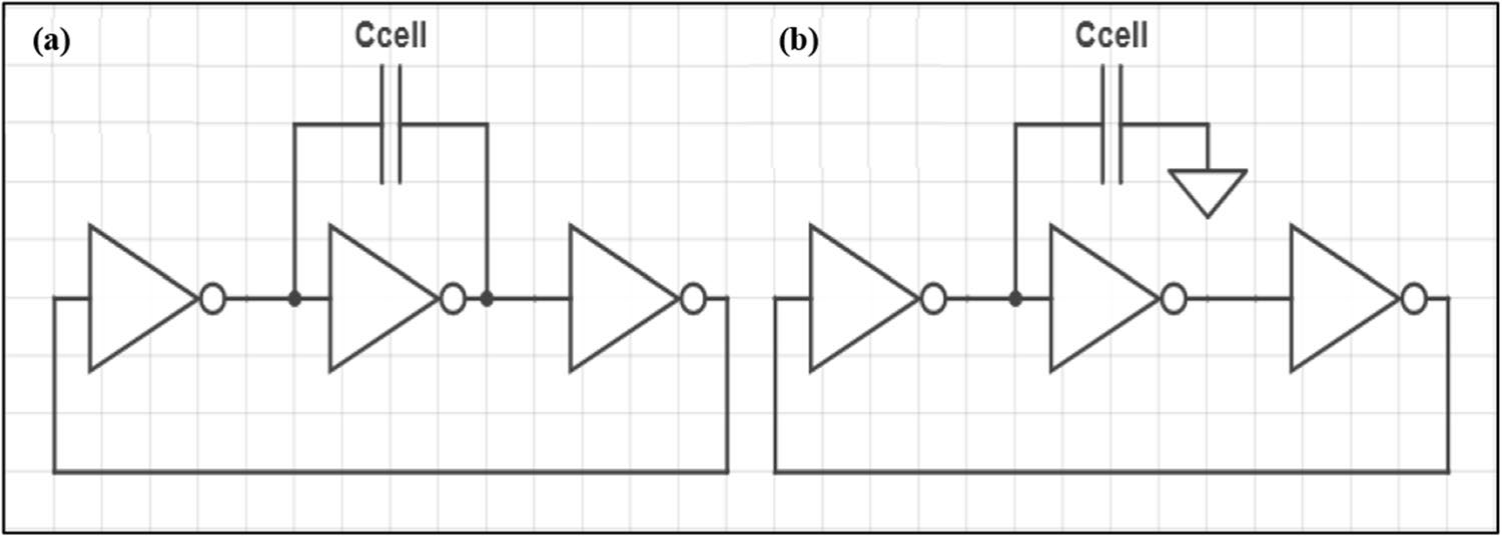
(**a**) Circuit diagram of miller capacitance configuration (MCC). (**b**) Circuit diagram of Grounded capacitance configuration (GCC).

The second subsystem of the proposed biochip is the pixel readout circuit (PRC) which exists for every capacitive sensor in the CSM. When a sample cell is introduced to the CSM electrodes, a dielectric layer begins to form, increasing the electrodes' capacitance^18^. The objective of the readout circuit is to utilize the capacitive change captured from the CSM's electrodes to locate and characterize the introduced cell. The location of the cell is identified by calculating the pixel with the greatest capacitive change. The measured capacitance value is also used to characterize whether it is a normal liver cell or HCC. The design of the PRC is composed of a three-stage ring oscillator implemented by three serially connected inverters. The CSM electrodes are connected to each readout circuit using two proposed configurations, the miller capacitance configuration (MCC) and the grounded capacitance configuration (GCC). The miller capacitance configuration, MCC, places the electrodes across one inverter stage, as shown in Fig. 4a. In contrast, the grounded capacitance configuration, GCC, places both electrodes between two inverter stages, as shown in Fig. 4b. The performance of both architectures will be evaluated in the results section. For both configurations, the main operating principle of the pixel readout circuit is a capacitance-to-frequency transduction mechanism. The ring oscillator design offers several advantages, such as high noise immunity and a dynamic output range because it is a digital output signal with a varying frequency^25^.

This functionality is achieved by modulating the three-stage ring oscillator's output frequency using the cell's capacitive change, as shown in Fig. 4a,b.

Assuming equal high to low and low to high inverter transitions, the output frequency, ${\varvec{f}}$, of the ring oscillator can be linked to each inverter stage delay using the following equation:

2 $$f=\frac{1}{tp1+tp2+tp3}$$

where $tp1, \; tp2, \; tp3$ are the delay of inverter stages 1, 2, and 3, respectively.

Equation (3) presents a first-order analysis of the delay of a CMOS inverter, $tp$, with a load capacitance, $Cl$, and assuming equal PMOS and NMOS on-resistance's, $Ron$^26^.

3 $$tp=0.69*Ron*Cl$$

Equations (2) and (3) illustrate the inverse relationship between the load capacitance of an inverter and the sensor's output frequency.

In both configurations, the load capacitance, $Cl$, of the first inverter stage is affected by the introduced cell capacitance, $Ccell$. The sensitivity of the pixel readout circuit is defined as the change in output frequency due to the capacitance change introduced by $Ccell$. The results section carries out a sensitivity comparison between the MCC and GCC for different capacitance values. In MCC, $Ccell$ is charged and discharged simultaneously, which effectively increases the load capacitance and accordingly decreases the output frequency^25^. However, in GCC, $Ccell$ is periodically charged and discharged at separate instances, and therefore, MCC is expected to exhibit a greater sensitivity to changes in $Ccell$ than GCC.

MCC and GCC are designed and simulated on Virtuoso Analog Design Environment (ADE)—Cadence. The libraries used are “tsmc13rf” for the 130 nm transistors and “analoglib” for the supply voltage of 1 V and ideal capacitance used to model the cell.

In series, the ring oscillator is composed of three identical CMOS inverters, NMOS and PMOS transistors, and is the same for the MCC and GCC models. In order to achieve a high base frequency at the output, the inverters are sized with minimum channel length and large width; additionally, the PMOS has sized a factor greater than the NMOS in order to achieve a near 50% duty cycle. PMOS length and width are 130 nm and 8450 nm, respectively, whereas PMOS length and width are 130 nm and 1300 nm.

In order to model the effect of adding a biological cell between the biochip's electrodes, an ideal capacitance is added to both the GCC and MCC models, as shown in Fig. 4a,b.

For each model, this capacitance is varied, and the corresponding output frequency of the circuit is calculated using the built-in frequency calculator function in ADE.

In the next sections, The MCC is simulated with PVT variations to validate the sensor's ability to distinguish between normal liver cells and HCC; the varied parameters and results are presented in Fig. 7c. Moreover, an eye diagram is performed in order to measure the impact of oscillator noise in terms of SNR and jitter on the output frequency.

A novel algorithm is used to represent the sensors' reading at different positions of a biological cell and predict the cell's shape using MATLAB R2022a. Firstly, the readings of all sensors are represented by a dynamic 3D plot versus time (the positions of sensors in the XY plane and the measured capacitance of each sensor in the z-direction). Secondly, the generated frames are analyzed to predict the shape of the tested cell at different positions. However, the detailed procedures for representing CSM readings and predicting the tested cell's shape are further discussed in the following section.

## Results

This section presents the results of both the FEM and Virtuoso ADE simulations of the CSM and the PRC. In addition, the results of the novel algorithm to animate and predict the shape and location of the tested biological cells are discussed in order to evaluate the proposed biochip's ability to track and characterize the microbiological cell.

**Figure 5:**
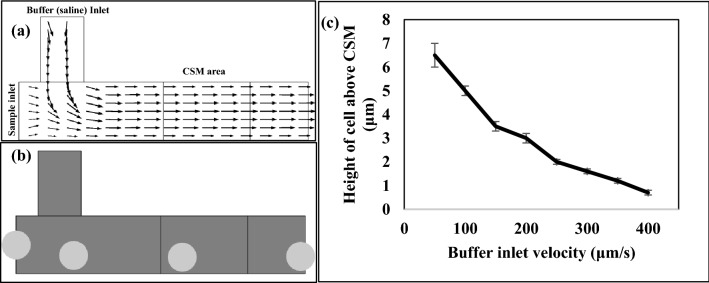
(**a**) The streamlines of the fluid velocity within the microfluidic chamber due to sample inlet velocity equal to 50 μm/s and buffer inlet velocity 150 μm/s. (**b**) The particle tracing of dragging tested cells to be close to CSM. (**c**) The effect of the buffer inlet velocity on the height of tested cells above CSM.

As illustrated in Fig. 5, the microfluidic chamber is intended to keep the tested cells at a specific height to increase the sensitivity of the proposed platform.

The impact of adding another inlet to adjust the height of the injected tested cells through the primary inlet is shown in Fig. 5. Figure 5c depicts that the higher the buffer inlet velocity, the closer the cells are to the CSM. Consequently, by regulating the buffer inlet velocity, we can keep the cells at a consistent height with a tiny error of 0.1.

**Figure 6:**
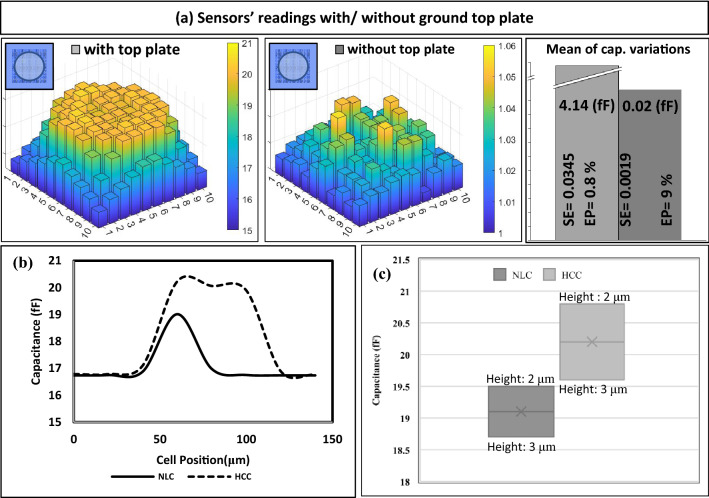
(**a**) A comparison between the sensor's reading using two configurations with/without a ground top plate in terms of the mean of capacitance variations (capacitance of HCC suspended in saline—capacitance of saline) (SE: standard error, and EP: Error percentage), (**b**) Sensor E5′ readings of NLC and HCC versus cell position in the x-direction at height 2.5 µm. (**c**) CSM readings at different heights of NLC and HCC above the CSM (Height (H): 2.5 ± 0.5 µm) in z-direction.

Different configurations of CSM are simulated in order to determine the most effective configuration for sensing the cell capacitance, as shown in Fig. 6a. However, an algorithm using MATLAB succeeded in visualizing the readings of CSM, as shown in Fig. 6a. As shown in Fig. 6a, the height and the color of each bar, which represents each sensor reading, change by changing the location of the cell, where green indicates that the cell is far, orange indicates that the cell is nearby, and yellow means that the cell is above the sensor. As indicated in Fig. 6a, there is a significant improvement in the sensitivity of the sensors in the tested cell when using a ground top plate with a very low error rate (0.8%) and large capacitance variation (4.14 fF) due to presence of biological cell (HCC), compared to not utilizing a ground top plate, which results in a substantial disparity between the sensors readings (9%). However, the large capacitance variation allows straightforward biological cell detection by the readout circuit. In addition, different conditions of tested biological cells are simulated to determine the sensitivity of CSM under different conditions of biological cells (e.g., different types, sizes, positions in the x-direction, heights in the z-direction) as shown in Fig. 6b,c. Figure 6b depicts the capacitance of animated NLC and HCC at various places in the x-direction.

As shown in Fig. 6b, It is possible to identify the size and type of the cell by the width and height of the pulse of the measured capacitance of a single sensor (E5). Figure 6b also shows that the larger the tested cell size, the larger the measured capacitance by a sensor of CSM. In the vertical direction of the CSM plane, different heights of the biological cell above one of the sensors are measured by CSM, as shown in Fig. 6c. This comparison is preferred to study the effect of the distance in the Z-direction on the readings. The results indicated that with a probability of 20% changes (2.5 ± 0.5 µm) in the height of each cell, there is still a difference between the measured capacitances of NLC (19.1 ± 0.4) and HCC (20.23 ± 0.6). Figure 6c also shows that the larger the height of the tested cell size, the smaller the measured capacitance by a sensor of CSM. Therefore, the cell is preferably kept at a certain height to the sensor during the measurement to ensure maximize the sensitivity. Additionally, the presence of the top ground plate has the added advantage of reducing the space in which the cell will be present, which improves cell-to-sensor proximity.

Table 1 presents the extracted capacitance values of air, saline, NLC, and HCC from the CSM COMSOL Multiphysics model. In the case of air and saline, the average and standard deviation are computed using all of the sensors, but in the case of cells, it is calculated using only the sensors that sensed the cell, and these are 2 × 2 sensors for NLC and 6 × 6 sensors for HCC.

**Table 1:** The estimated capacitance of biological samples using FEM.

Cell	Air	Saline	Saline + NLC	Saline + HCC
Capacitance (fF)	0.23	16.5	19.1	20.23
Standard deviation (std)	3.06E−3	0.285	0.0179	0.188
Standard error of sensors $(\frac{\mathrm{Std}}{\sqrt{\mathrm{N}}})$	± 3.06E−4	± 2.85E−2	± 8.99E−3	± 3.14E−2
Standard error at different heights of cells	–	–	± 0.4	± 0.6
Cumulative errors (fF)	± 3.06E−4	± 2.85E−2	± 0.4009	± 0.60314

These extracted capacitance values will be used in testing the readout circuit to evaluate its characterization and sensing performance.

**Figure 7:**
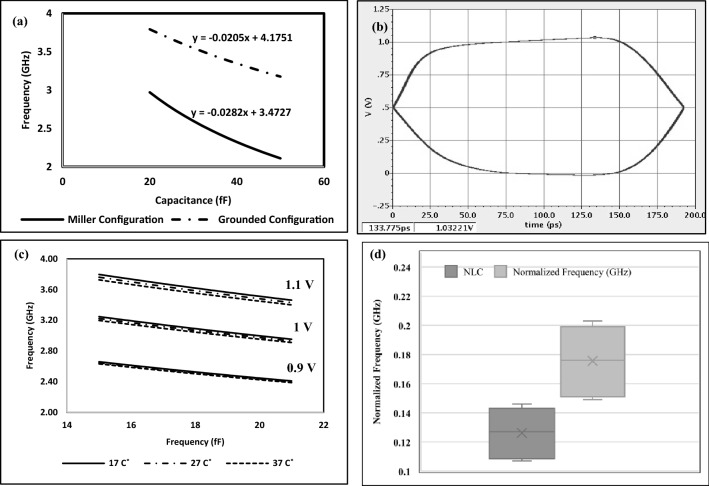
(**a**) The simulated output frequency of the pixel readout circuit versus the estimated capacitance values. (**b**) Eye diagram analysis of the output frequency signal for Saline (C = 16.5 fF). (**c**) the maximum positive and negative deviation in measurements of PVT variations (Voltage: 1 ± 0.1 V, Temperature: 27 ± 10 °C), and (**d**) Normalized (subtract saline values from NLC and HCC values) frequency values of the PVT study of HCC and normal liver cell with diameters 20, 50 µm that have capacitance equal 19.1 ± 0.4 and 20.2 ± 0.6 fF, respectively.

The MCC and GCC output frequencies for a single sensor are compared for different capacitance values extracted from COMSOL Multiphysics, as shown in Fig. 7a. The metric of interest is the slope of the output frequency as a function of capacitance as it presents the sensor's sensitivity. The results of the comparative experiment indicate that the MCC offers a higher sensitivity to capacitive changes than the GCC; therefore, the MCC is selected for all following simulations. The test procedure of the MCC is to first get a reference reading by inputting saline's capacitance into the simulation and recording the output frequency. The next step is to input the capacitance of the HCC or NLC and record the output frequency after subtracting the saline or reference measurement. Table 2 presents the results of testing the MCC using the capacitance values of saline (C = 16.5 fF). Moreover, the eye diagram plot of the output frequency for Saline (C = 16.5 fF) is presented in Fig. 7b. The jitter (the line thickness at the 0.5 V crossing point), SNR (eye height), duty cycle (eye corner), period, and output frequency, denoted by $Fout$, are calculated and presented in Table 2. Equation. four shows how the frequency Error due to jitter, $Ferror$, is calculated.

**Table 2:** Eye diagram parameters.

Parameter	Jitter (eye thickness) (psec)	Signal half period (psec)	Output frequency (GHz)	Frequency error from jitter (GHz)	SNR (eye height)	Duty cycle (eye corner)
Value	1.2	192	2.5997	0.0162	1	49.80%

4 $$Ferror=\left(\frac{Jitter}{Period}\right)*Fout$$

Figure 7c presents the maximum positive and negative deviation in measurements of PVT variations (Voltage: 1 ± 0.1 V, Temperature: 27 ± 10 °C). As shown in Fig. 7c, Variations in voltage have a greater effect on output frequency than temperature changes but with the same slope.

Figure 7d shows the normalized frequency of NLC and HCC by subtracting the output frequency of saline from the output frequency of NLC suspended in saline and HCC suspended in saline in order to reduce the impact of PVT variations on the sensor's characterization performance. These results demonstrate the sensor's ability to accurately characterize normal and abnormal cells in the presence of PVT variations. Due to the normalized measurement, any parasitic that can arise from routing are removed as they will common frequency offsets to both the saline and NLC/HCC measurements.

The frequency error due to jitter (0.0162 GHz) in Table 2 can be neglected because it is less than the difference between the normalized normal liver cell and HCC (0.106 GHz), as shown in Fig. 7d. Furthermore, the power consumption is 459.3 μW and was calculated by taking the average of the total power drawn from the Vdd.

**Figure 8:**
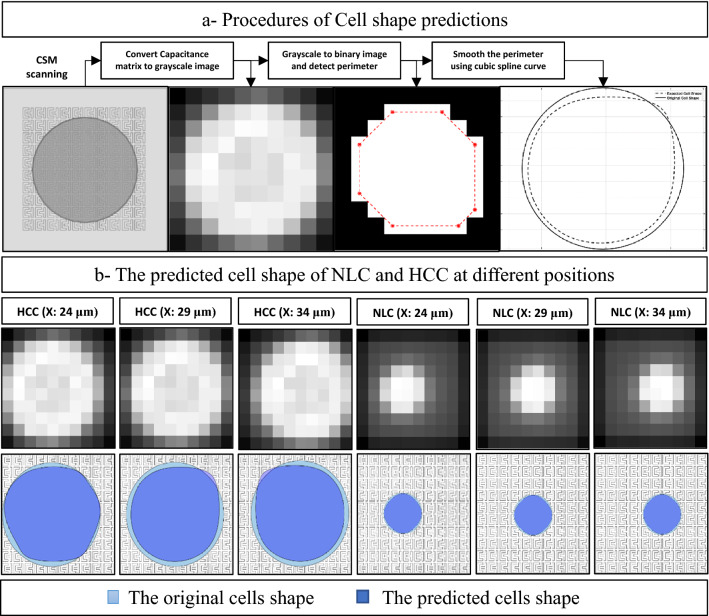
(**a**) The procedures of estimating the cell shape, (**b**) the shape estimation of NLC and HCC at different position shapes based on CSM readings from COMSOL Multiphysics.

As shown in Fig. 8a, A novel algorithm using MATLAB is developed to estimate the shape and location of the tested cell using many steps. First, the capacitance matrix is converted to a grayscale image using a built-in function (mat2gray). Then, the grayscale image is converted to a binary image using a threshold equal to the mean of capacitance values. The contour of the binary image is determined using a built-in function (bwperim, and convhull). The contour is smoothed using the cubic spline curve's built-in function (cscvn). The polygon's centroid is determined using a built-in function (centroid). The radius is determined by searching for the most frequent radius (mode) from an array of distances estimated using (Euclidean distance between each point on the polygon and the centroid).

A novel algorithm using MATLAB succeeded in estimating the shape of NLC and HCC using the readings of CSM at different cell positions, as shown in Fig. 8b. The sensors' readings at different positions of NLC and HCC are analyzed to model the shape and location of the tested cell. In Fig. 8b, the expected cell shape and location visualization are almost identical to the animated cell movement at each step. However, the proposed CSM estimation accuracy is 96.5% in estimating the radius and 98.1% in estimating the location of the tested HCC, while 97% in calculating the radius and 100% in estimating the location of the tested NLC.

Table 3 provides a performance comparison of various CMOS capacitive biosensors. Our research is the first to focus on shape prediction of the tested biological cells in biosensor arrays, whereas others solely deal with capacitance readings. In addition, we are interested in investigating and eliminating the impact of any faults that could occur due to the sensor matrix or the circuit. These faults include the height of the tested biological cells within the microfluidic channel, the sensors' sensitivity to the presence of biological cells, the influence of temperature and voltage variations, and the effect of parasitic capacitance.

**Table 3:** Comparison with other work using bipolar capacitive sensor.

Cell type	^11^	^14^	This work
Technology (μm)	0.35	0.25	0.13
Supply (V)	3.3	2.5	1.2
Power	300 mW	29 μW	459.3 μW
Readout method	Capacitance to frequency	Charging share	Capacitance to frequency
Sensors number	4 × 4	16 × 16	10 × 10
Sensor size (μm^2^)	30 × 30	14 × 16	5 × 5
Sensitivity	0.6 MHz/fF	55 mv/fF	46 MHz/fF
Input range (fF)	0.1–1	0.45–57	15–21
Microparticles	Ovarian cancer	epidermdis	HCC
Media	–	PBS	Saline
Capacitance value	100 aF	450 aF	20.2 fF
Cell shape prediction	No	No	yes

However, the impact of these errors has been canceled using the following: a ground top plate is added to enhance the sensors' sensitivity to the presence of biological cells. The microfluidic chamber is developed to keep the tested biological cells at a certain height. Using saline as a reference, the capacitance reading is normalized to remove the effect of the readout circuit errors such as parasitic capacitance, temperature changes, and voltage changes.

## Conclusion

A capacitive-based biochip is presented and discussed for characterizing and locating the biological cells. A FEM model of a matrix of 10 × 10 capacitive sensors is implemented using COMSOL Multiphysics 5.5. A ring oscillator-based readout circuit is simulated using Virtuoso Analog Design Environment—Cadence. Different configurations for measuring the biological cell's capacitance are tested to select the optimal configuration that maximizes sensitivity. The results prove that the top ground plate with default grounded sensors except the activated sensor is the ideal configuration for the CSM. The effect of the distance between the tested cells and the CSM plane on the capacitance readings is explored and discussed. The results indicate that the closer the cells are to the sensor, the higher the measured capacitance leading to better sensitivity, which is intuitively expected.

Several types and sizes of biological cells (e.g., NLC and HCC suspended in saline) are modeled and tested using the proposed CSM. The results of the measured capacitance and its correlated frequency from both models, CSM and the readout circuit prove the proposed architecture's ability to differentiate between different types and sizes of biological cells (NLC and HCC). A novel algorithm is developed using MATLAB R2022a in order to animate the simulated cells' movement and predict the shape and location of tested cells using the measured capacitances by CSM. The results indicate that the developed algorithm succeeded in estimating with almost a hundred percent accuracy the location and size of NLC and HCC. Also, this monitoring allows estimating additional features such as the speed and volume of the manipulated particles without external equipment such as an optical microscope. Finally, a completely integrated biochip for monitoring and identifying biological cells is developed utilizing a ring oscillator-based capacitive sensor matrix, which has numerous advantages such as efficiency, small size, low cost, and eliminates the need for sample labeling and external equipment. This work shows promising results as a proof of concept for a biochip suitable for characterization and imaging. Future work will include using smaller technologies for improved sensitivity and a fully fabricated chip for testing real biological cells.

## Data Availability

The data supporting this study's findings are available from the corresponding author upon reasonable request.
